# Supplementary material for: A data assimilation method to track excitation-inhibition balance change using scalp EEG
Source: Commun Eng. 2023 Dec 16;2:92. doi: 10.1038/s44172-023-00143-7 (PMC10956076; doi:10.1038/s44172-023-00143-7)
Supplement: Supplementary file 1 — Supplemental Information [file 44172_2023_143_MOESM1_ESM.pdf]

Supplementary information:

A data assimilation method to track excitation-inhibition balance change using scalp EEG

Hiroshi Yokoyama[1, 2*], Keiichi Kitajo[1, 3*]

1. Division of Neural Dynamics, Department of System Neuroscience, National Institute for Physiological Sciences, 38 Nishigonaka, Myodaiji, Okazaki, Aichi, 444-8585, JAPAN
2. Data Science and AI Innovation Research Promotion Center, Shiga University, 1-1-1 Baba, Hikone, Shiga, 522-8522, Japan.
3. Physiological Sciences Program, Department of Advanced Studies, Graduate University for Advanced Studies (SOKENDAI), 38 Nishigonaka, Myodaiji, Okazaki, Aichi, 444–8585, Japan

*Corresponding author(s). E-mail(s): yokoyama@nips.ac.jp ; kkitajo@nips.ac.jp

Supplementary Methods:

S1. *Variational inference for the Kalman Filter*

As stated in the main manuscript, to consider the effects of unknown or nonstationary observation noise, we applied a variational Bayesian noise adaptive approach for the Kalman Filter (vbKF) [1-5]. First, we will explain the mathematical details of the noise adaptive algorithm. For the sake of simplicity, we will here give a detailed description of the algorithm for the vbKF in a Gaussian linear case. A discrete-time linear state space model is defined using the following equations:

$$x_{t+1} = f(x_t) + \zeta_t = Fx_t + \zeta_t, \quad \zeta_t \sim \mathcal{N}(0, Q),$$

$$y_t = h(x_t) + w_t = Hx_t + w_t, \quad w_t \sim \mathcal{N}(0, \eta^{-1}R), \eta_t \sim \mathcal{G}(\alpha, \beta).$$

We here assumed that the probability of the observation noise follows the normal-gamma probability density. Furthermore, observation y_t and state variable x_t follow the multivariate Gaussian distribution $\mathcal{N}(Hx_t, \eta_t^{-1}R)$ and $\mathcal{N}(m_t, P_t)$, respectively. In such a case, if the state variable x_t and noise scaling parameter η_t are independent, the joint posterior probability density for these variables can be estimated with the variational Bayesian approximate, as below [2]:

$$p(x_t, \eta_t | y_{1:t}) = p(y_t, x_t, \eta_t | y_{1:t-1}) \approx q(x_t) q(\eta_t).$$

The variational Bayesian approximation can be solved by minimizing the Kullback–Leibler (KL) divergence between the approximated distribution $q(x_t)q(\eta_t)$ and the exact posterior $p(y_t, x_t, \eta_t | y_{1:t-1})$, as below [2]:

$$\mathrm{KL}\big(q(x_t)q(\eta_t) \; || \; p(y_t, x_t, \eta_t | y_{1:t-1})\big) = \int \left\{ q(x_t)q(\eta_t) \times \ln\left(\frac{q(x_t)q(\eta_t)}{p(y_t, x_t, \eta_t | y_{1:t-1})}\right)\right\} dx_t d\eta_t.$$

Minimizing the right term of the above equation with respect to the probability $q(x_t)$, the solution of updating rule of the probability density for x_t is given as the following equation:

$$\begin{aligned}
\ln q(x_t) &= \langle \ln p(y_t, x_t, \eta_t | y_{1:t-1}) \rangle_{\eta_t} + const. \\
&= \langle \ln\{p(y_t | x_t, \eta_t)\, p(x_t | y_{1:t-1})\} \rangle_{\eta_t} + const. \\
&= -\frac{1}{2} x_t^T \left(P_t^{-1} + \langle \eta \rangle_\eta^{-1} R^{-1} H^T H\right) x_t - \left(\langle \eta_t \rangle_{\eta_t} R^{-1} y_t^T H + m_t^T P_t^{-1}\right) x_t + const. \\
&= \ln \mathcal{N}(x_t | m_{new}, P_{new}) + const.
\end{aligned}$$

This means that the parameters m_{new} and P_{new} can be calculated as follows:

$$P_{new} = \left(P_t^{-1} + \langle \eta_t \rangle_{\eta_t} R^{-1} H^T H\right)^{-1} = P_t - P_t H^T \left(\langle \eta_t \rangle_{\eta_t}^{-1} R + H P_t H^T\right)^{-1} H P_t,$$

$$m_{new} = P_{new}^{-1}\left(\langle \eta_t \rangle_{\eta_t} R^{-1} y_t^T H + m_t^T P_t^{-1}\right) = m_t + PH^T\left(\langle \eta_t \rangle_{\eta_t}^{-1} R + HPH^T\right)^{-1}(x_t - Hm_t^T).$$

Note that $\langle\cdot\rangle_{\eta_t}$ indicates the expectation operator under the variable η_t.

In the same manner, the solution of $q(\eta_t)$ is also given, as follows:

$$\begin{aligned}
ln\, q(\eta) &= \langle \ln p(y_t, x_t, \eta | y_{1:t-1}) \rangle_{x_t} + const. \\
&= \langle \ln\{p(y_t | x_t, \eta_t)\, p(\eta | y_{t-1})\} \rangle_{x_t} + const. \\
&= \left\langle \left(\alpha_t + \frac{N}{2} - 1\right) \ln|\eta_t| - \left[\beta_t + \frac{1}{2}(y_t - Hx_t)^T R^{-1} (y_t - Hx_t)\right] \eta_t \right\rangle_{x_t} + const. \\
&= \ln \mathcal{G}(\eta_t | a_{new}, b_{new}) + const.
\end{aligned}$$

Note that $\langle\cdot\rangle_{x_t}$ indicates the expectation operator under the variable x_t. The parameters a_{new} and b_{new}

can be calculated as follows:

$$\alpha_{new} = \alpha_t + \frac{N}{2} ,$$

$$\beta_{new} = \beta_t + \frac{1}{2}\langle (y_t - Hx_t)^T R^{-1} (y_t - Hx_t)\rangle_{x_t} = \beta_t + \frac{1}{2}\frac{\{\|y_t - H^T m_t\|^2 + H^T P_t H\}}{R} .$$

The expectation of parameter η_t (i.e., $\langle\eta_t\rangle_{\eta_t}$) can be described as follows:

$$\langle\eta_t\rangle_{\eta_t} = \frac{\alpha_{new}}{\beta_{new}}.$$

S2. *Inequality constraint for Kalman Filter*

In this section, we will explain the details of the state estimation in KF with an inequality constraint. In the context of the study on the control theory in robotics, various approaches of imposing the constraint in state estimation for the KF scheme have been proposed [6, 7]. Among these, we chose to apply the estimation projection approach with inequality constraints [6-9] in our proposed method. In this approach, if the state variables x_t follow the probability density $x_t \sim p(x_t|x_{t-t}) = \mathcal{N}(m_t, P_t)$ with the constraint $Dx_t \leq d_U$, the KF algorithm estimates the value of m_t and P_t so as to solve the following problem:

$$\underset{x}{\mathrm{argmax}}: \ln p(x_t|x_{t-1}) \;\;\Rightarrow\;\; \mathrm{argmin}: (x_t - m_t)P_t^{-1}(x_t - m_t),$$

$$\text{s.t., } Dx_t \leq d_U.$$

This problem can be solved using a Lagrange multiplier method, as follows:

$$L = (x_t - m_t)P_t^{-1}(x_t - m_t) + 2\lambda^T(Dx_t - d_U)$$

The solution is given as follows:

$$x_t = m_t - P_t D^T (D P_t D^T)^{-1} (D m_t - d_U).$$

In the same manner, we can solve the solution in the case of the constraint $Dx_t \geq d_L$, as follows:

$$x_t = m_t - P_t D^T (D P_t D^T)^{-1} (D m_t - d_L).$$

In the current study, to estimate the state and parameter using the EnKF scheme while considering parameter constraints so as to generate the periodic signal in the NM model, we applied the following interval constraint:

$$d_L \;\leq\; D\,x_t \leq\; d_U, \text{ where } x_t = [v_t, \theta]^T = \left[[v_0, v_1, \dots, v_5], [A, a, B, b, p]\right]^T.$$

Note that parameters $D, d_L,$ and d_U followed with $D = [\mathbb{O}^{5\times6} \;\; \mathbb{I}^{5\times5}]$, $d_L = [0.01, 5.00, 0.01, 5.00, 120]^T$, and $d_U = [100.00,\ 200.00,\ 100.00,\ 200.00,\ 320.00]^T$; that is, such interval constraints were set to restrict the range of values in the model parameters $A, a, B, b,$ and p. In particular, interval constraints for the parameter p in the NM model were selected so that the estimated value of p was satisfied with the interval $120 \leq p \leq 320$. This was chosen based on the study by Jansen and Rit (1995) [10], which showed that the alpha-like periodic simulated signal was observed in the NM model when the external input p was distributed in the range of $120 \leq p \leq 320$. An interval constraint for the inverse of time constant a in excitatory post-synaptic potential (PSP) was chosen as $5 \leq a \leq 200$ to satisfy $5 \leq \tau_e[\text{ms}] \leq 200$, where $\tau_e = a^{-1}$. Also, the constraint for the inverse of time constant b in inhibitory PSP

was chosen to satisfy $5 \le \tau_i[ms] \le 200$, where $\tau_i = b^{-1}$. The parameters a and b were selected so that the NM model can generate delta to beta rhythms based on the report by David and Friston (2002) [11].

Supplementary References

1. Dong, Z. & Song, T. Variational inference of Kalman filter and its application in wireless sensor networks. *International Journal of Distributed Sensor Networks* **2013** (2013). https://doi.org/10.1155/2013/106434 .
2. Sarkka, S. & Nummenmaa, A. Recursive noise adaptive Kalman filtering by variational Bayesian approximations. *IEEE Transactions on Automatic Control* **54** (3), 596–600 (2009). https://doi.org/10.1109/TAC. 2008.2008348 .
3. Stroud, J. R. & Bengtsson, T. Sequential state and variance estimation within the ensemble Kalman filter. *Monthly Weather Review* **135** (9), 3194–3208 (2007). https://doi.org/10.1175/MWR3460.1 .
4. Wang, G. Y. & Guan, B. L. Fuzzy adaptive variational Bayesian unscented Kalman filter. *Journal of Information Hiding and Multimedia Signal Processing* **6** (4), 740–749 (2015) .
5. Wang, S. Y., Yin, C., Duan, S. K. & Wang, L. D. A Modified Variational Bayesian Noise Adaptive Kalman Filter. *Circuits, Systems, and Signal Processing* **36** (10), 4260–4277 (2017). https://doi.org/10.1007/ s00034-017-0497-6 .
6. Simon, D. & Chia, T. L. Kalman filtering with state equality constraints. *IEEE Transactions on Aerospace and Electronic Systems* **38** (1), 128–136 (2002). https://doi.org/10.1109/7.993234 .
7. Simon, D. Kalman filtering with state constraints: a survey of linear and nonlinear algorithms. *IET Control Theory Applications* **4** (8), 1303–1318 (2010). https://doi.org/10.1049/iet-cta.2009.0032 .
8. Luzar, M., Czajkowski, A., Witczak, M. & Korbicz, J. Actuators and sensors fault diagnosis with dynamic, state-space neural networks. *2012 17th International Conference on Methods and Models in Automation and Robotics, MMAR 2012* (August), 196–201 (2012). https://doi.org/10. 1109/MMAR.2012.6347889 .
9. Wang, D., Chen, Y. & Cai, X. State and parameter estimation of hydrologic models using the constrained ensemble Kalman filter. *Water Resources Research* **45** (11), 1–13 (2009). https://doi.org/10.1029/ 2008WR007401 .
10. Jansen, B. H. & Rit, V. G. Electroencephalogram and visual evoked potential generation in a mathematical model of coupled cortical columns. *Biological Cybernetics* **73** (4), 357–366 (1995). https://doi.org/10.1007/ BF00199471 .
11. David, O. & Friston, K. J. A neural mass model for MEG/EEG: Coupling and neuronal dynamics. *NeuroImage* **20** (3), 1743–1755 (2003). https: //doi.org/10.1016/j.neuroimage.2003.07.015 .
